# The Revised Human‐Centered Design of MyCog 2.0:Validation of an App‐Based Self‐Administered Cognitive Screening Tool for Older Adults

**DOI:** 10.1002/alz70863_110678

**Published:** 2025-12-23

**Authors:** Stephanie Ruth Young, Julia Yoshino‐Benavente, Richard C. Gershon, Cindy J. Nowinski, Michael S Wolf

**Affiliations:** ^1^ Northwestern University Feinberg School of Medicine, Chicago, IL USA; ^2^ Northwestern University, Chicago, IL USA

## Abstract

**Background:**

Primary care visits offer an important opportunity for early detection of cognitive impairment (CI) in adults over age 65, yet less than half of patients with any CI are identified in primary care settings. To address barriers to screening in primary care and improve early detection rates, the NIH funded MyCog, a self‐administered, tablet‐based cognitive screener that integrates with an electronic health record and triggers appropriate decision‐making support. The MyCog app is currently used in studies in three large health systems, Northwestern Medicine, Oak Street Health, and Access Community Health Network, funded by two large NIH grants (U01NS105562 and R01AG069762). These clinical sites have provided substantial feedback on the app resulting in meaningful improvements to the user experience and app interface. We present evidence from the construct validation of the upgraded MyCog app, and discuss usability implications in our clinical sites.

**Method:**

Data from a community sample (n = 200; mean age = 73 years) who had completed the upgraded MyCog were compared to an age‐matched sample who had previously completed the original MyCog. Internal consistency and construct validity were evaluated via confirmatory factor analysis. Bayesian differential item functioning was employed to evaluate equivalence between MyCog and MyCog 2.0.

**Result:**

Internal consistency was high for executive function (rsb = .92) and episodic memory tests (ωt = .84). A two‐factor model showed excellent fit (χ²(8) = 6.73, *p* = .566, CFI = 1.000, TLI = 1.014, RMSEA = 0.000 (90% CI [0.000, 0.077], *p* = .813), SRMR = 0.030) significantly better than a one factor model ((Δχ²(1) = 12.86, *p* < .001; ΔAIC = 10.8; ΔBIC= 7.6), suggesting the tests measure related but distinct constructs as hypothesized. Differential item functioning between the two test versions was not observed for episodic memory performance or executive functioning accuracy; however, response time on five executive function items differed.

**Conclusion:**

Findings support MyCog as a reliable and valid self‐administered cognitive screener designed specifically for ease of use among older adults. Usability improvements did not sacrifice psychometric validity of the tool and support MyCog's continued use in clinics. Clinical validation studies are forthcoming.

